# Antimicrobial exposure and the risk of delirium in critically ill patients

**DOI:** 10.1186/s13054-018-2262-z

**Published:** 2018-12-12

**Authors:** Jessica J. Grahl, Joanna L. Stollings, Shayan Rakhit, Anna K. Person, Li Wang, Jennifer L. Thompson, Pratik P. Pandharipande, E. Wesley Ely, Mayur B. Patel

**Affiliations:** 1 Department of Pharmaceutical Services, Vanderbilt University Medical Center, 1211 Medical Center Drive, Nashville, TN 37212 USA; 2 Critical Illness, Brain dysfunction, Survivorship (CIBS) Center, 2525 West End Avenue, Nashville, TN 37232 USA; 3 Vanderbilt University School of Medicine, 2215 Garland Avenue, Nashville, TN 37212 USA; 4 Division of Infectious Diseases, Department of Medicine, Vanderbilt University Medical Center, 1161 21st Avenue S, Nashville, TN 37232-2650 USA; 5 Department of Biostatistics, Vanderbilt University School of Medicine, 2525 West End Avenue, Nashville, TN 37232 USA; 6 Division of Anesthesiology Critical Care Medicine, Department of Anesthesiology, Vanderbilt University Medical Center, 1211 21st Avenue S, Nashville, TN 37212 USA; 7 Center for Health Services Research, Vanderbilt University Medical Center, 1215 21st Avenue S, Nashville, TN 27232-8300 USA; 8 Geriatric Research, Education and Clinical Center Service, Department of Veterans Affairs Medical Center, Tennessee Valley Health Care System, 1310 24th Avenue S, Nashville, TN 37212 USA; 9 Division of Pulmonary and Critical Care Medicine, Department of Medicine, Vanderbilt University Medical Center, 1161 21st Avenue S, Nashville, TN 37232-2650 USA; 10 Division of Trauma and Surgical Critical Care, Departments of Surgery, Neurosurgery, and Hearing & Speech Sciences, Section of Surgical Sciences, Vanderbilt Brain Institute, Vanderbilt University Medical Center, 1211 Medical Center Drive, 404 Medical Arts Building, Nashville, TN 37212 USA

**Keywords:** Delirium, Encephalopathy, Neurotoxicity, Antibiotics, Critical care

## Abstract

**Background:**

Prior retrospective cross-sectional work has associated antimicrobials with a non-specific phrase: encephalopathy without seizures. The purpose of this study is to determine whether different classes of antimicrobials have differential associations with the daily risk of delirium after critical illness is adjusted for.

**Methods:**

Our study was a nested cohort that enrolled non-neurological critically ill adults from a medical or surgical intensive care unit (ICU) with daily follow-up to 30 days. Our independent variable was exposure to previous-day antimicrobial class: beta-lactams (subclasses: penicillins, first- to third-generation cephalosporins, fourth-generation cephalosporins, and carbapenems), macrolides, fluoroquinolones, and other. We adjusted for baseline covariates (age, comorbidities, cognition scores, sepsis, and mechanical ventilation), previous-day covariates (delirium, doses of analgesics/sedatives, and antipsychotic use), and same-day covariates (illness severity). Our primary outcome of delirium was measured by using the Confusion Assessment Method for the ICU. A daily delirium logistic regression model was used with an ICU time-restricted sensitivity analysis including daily adjustment for sepsis and mechanical ventilation.

**Results:**

Of 418 ICU patients, delirium occurred in 308 (74%) with a median of 3 days (interquartile range 2–6) among those affected and 318 (76%) were exposed to antimicrobials. When covariates and ICU type were adjusted for, only first- to third-generation cephalosporins were associated with delirium (logistic regression model odds ratio (OR) = 2.2, 95% confidence interval (CI) 1.28–3.79, *P* = 0.004; sensitivity analysis OR = 2.13, 95% CI 1.10–4.10, *P* = 0.024).

**Conclusions:**

First-, second-, and third-generation cephalosporins doubled the odds of delirium after baseline co-morbidities, ICU type, the course of critical care, and other competing antimicrobial and psychotropic medication risks were adjusted for. We did not find an association between delirium and cefepime, penicillins, carbapenems, fluoroquinolones, or macrolides.

**Electronic supplementary material:**

The online version of this article (10.1186/s13054-018-2262-z) contains supplementary material, which is available to authorized users.

## Background

Critically ill patients are commonly initiated on antimicrobial therapy for infection with one or more agents on the basis of physiologic, microbiologic, and pharmacologic factors [1]. Antimicrobial exposure may represent a risk for antibiotic-associated encephalopathy [2–5] or the other synonymous and broadly used term for encephalopathy (that is, delirium) [2, 3, 6, 7]. Delirium in hospitalized patients is a strong independent predictor of mortality, increased hospital length of stay, long-term cognitive impairment, cost of care, and subsequent hospitalizations [2, 8, 9].

The associations between antimicrobials and delirium have been limited by cross-sectional approaches and examination of only certain antibiotic classes, such as cephalosporins (for example, cefepime) [2, 3, 5, 10–13], fluoroquinolones [14, 15], and macrolides [2, 4], without accounting for confounders related to baseline comorbidities, sepsis, severity of illness, exposure to analgesics, sedatives, and other antimicrobial possibilities (for example, simultaneous exposure or no exposure) [2]. Importantly, there are many risk factors for delirium per intensive care unit (ICU) patient, including ongoing infection, severity of illness, older age, and baseline cognitive impairment [16–19]. Furthermore, no study of antimicrobials and acute brain dysfunction has used a reliable or validated tool for the outcome of delirium.

The purpose of this study is to determine whether there is an independent association between antimicrobial class exposure in critically ill patients and the daily risk of delirium, measured by using a valid and reliable tool repeatedly over time. Accounting for daily ICU risks, we hypothesized that different classes of antimicrobials would have differential associations with the daily risk of delirium.

## Methods

### Study design and population

The Vanderbilt University Institutional Review Board approved the study protocol. Described in detail elsewhere [8], the parent BRAIN-ICU Study included adults who received treatment in the medical or surgical ICU for respiratory failure or shock (or both) between March 2007 and May 2010. This parent cohort excluded patients if they met one or more of the following criteria: recent substantial critical illness requiring ICU admission, conditions that would make assessments for delirium unreliable (for example, deafness and blindness), conditions that would prohibit long-term follow-up owing to active substance abuse, psychotic disorder, homelessness, or residence 200 miles or more from the enrolling center, life expectancy less than 24 h, lack of informed consent, known or suspected severe neurologic disease such as from an anoxic or traumatic brain injury, and severe dementia. As previously reported, significant pre-existing cognitive impairment was excluded by using the combination of the Short Informant Questionnaire on Cognitive Decline in the Elderly (IQCODE) [20] score of at least 3.3 and the Clinical Dementia Rating score of more than 2.0 [8, 21, 22]. Pregnant patients, prisoners, and patients younger than 18 years of age were also excluded from the parent cohort.

For this current investigation, additional inclusion criteria consisted of BRAIN-ICU patients restricted to enrollment at a single hospital, Vanderbilt University Medical Center, with available medication administration records from time of enrollment to ICU discharge, and additional exclusion criteria consisted of those without medication records and those who died or withdrew within the first 48 h.

### Risk factors for delirium

The medication-administration record was used to collect information from the time of enrollment and throughout the ICU stay. Antimicrobial exposure was categorized as a previous-day exposure in the first model and total days of delirium during antimicrobial exposure in the second model. Antimicrobials were divided into four non-mutually exclusive groups: beta-lactams (subclasses: penicillins, first- through third-generation cephalosporins, cefepime, and carbapenems), macrolides, fluoroquinolones, and other while in the ICU. Antimicrobial exposure was assessed on a daily basis throughout ICU stay, and antimicrobial-free days were included in the statistical analysis in order to account for differences in total antimicrobial exposure during the study period. To avoid model overfitting, we created an “other” antimicrobial class, which included vancomycin, antifungals, antivirals, metronidazole, aminoglycosides, linezolid, sulfamethoxazole-trimethoprim, antiretrovirals, clindamycin, rifampin, doxycycline, tigecycline, dapsone, pentamidine, isoniazid, and ethambutol.

All other covariates were chosen *a priori* on the basis of clinical judgment and previous research, owing to their expected associations with antimicrobial exposure (independent variable) and with delirium (dependent variable) and thus their potential to be confounders. Baseline covariates included age, mechanical ventilation status, presence of severe sepsis, ICU type, Charlson Comorbidity Index, and Short IQCODE [8, 20, 23]. Previous-day covariates consisted of delirium, total daily dose of analgesics and sedatives (benzodiazepine, propofol, opiate, and dexmedetomidine), and antipsychotic use (typical antipsychotic [for example, haloperidol] and atypical antipsychotic [for example, quetiapine and olanzapine]). Analgesic and sedative drug doses were transformed with the use of their cube root to reduce the influence of extreme outliers. Same-day covariates consisted of modified Sequential Organ Failure Assessment (SOFA) score [24]. As previously done, the modified SOFA score was used and excluded the neurological components since coma was accounted for in all statistical models to determine primary outcome [8, 23]. This daily modified SOFA score was incorporated into statistical analyses in order to account for severity of illness, including degree of renal dysfunction, which is a risk factor for developing central nervous system toxicity and delirium in patients.

### Outcomes

Trained research personnel evaluated patients for delirium and level of consciousness daily until hospital discharge. Delirium was assessed with the utilization of the Confusion Assessment Method for the ICU (CAM-ICU) twice daily in the ICU and once daily on the ward [25, 26]. As previously described and in conjunction with structured evaluations for agitation and sedation [27], mental status on each day was classified as normal, delirious, or comatose [8, 21–23, 28]. Coma was defined as a Richmond Agitation Sedation Scale score of −4 or −5. The day of discharge was assigned a mental status of normal. All records prior to death or discharge were included for the longitudinal analysis.

### Statistical analysis

To test the associations between antimicrobial use and delirium, we used a multivariable adjusted regression model. To account for dependency between repeated measures [29] and longitudinal change in any mental status to delirium (that is, delirium versus normal/coma) in our multivariable adjusted model, we applied a logistic regression with cluster sandwich covariance estimator. Our second main model employed a proportional odds logistic regression model to explore the outcome of total days of delirium for each patient included in the cohort study as a function of total antimicrobial exposure.

Continuous variables were summarized as median with interquartile range (IQR). Categorical variables were summarized as a number with percentage (n, %). Multivariable analyses were reported with odds ratios (ORs) and 95% confidence intervals (CIs). Data and associated electronic materials were stored in a password-protected Research Electronic Data Capture (REDCap) database [30]. All analysis was performed by using statistical software R version 3.3.0 (R Development Core Team Vienna, Austria; https://www.r-project.org).

## Results

**Fig. 1:**
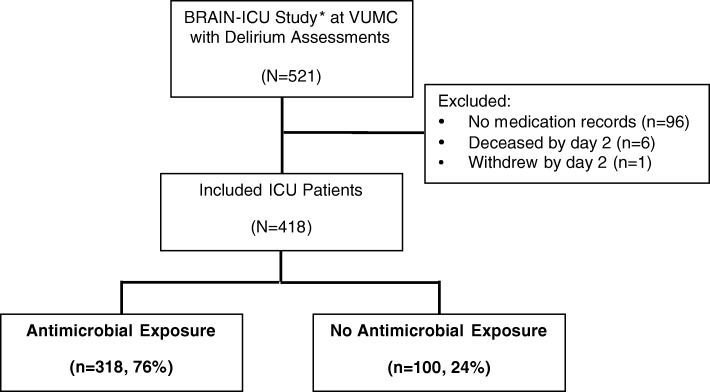
Cohort eligibility for antimicrobial exposures among critically ill patients with delirium assessments

Of the 521 patients enrolled at a single center within the BRAIN-ICU Study, 418 patients met our inclusion criteria. Exclusion criteria and exposure to antimicrobials in this ICU cohort with delirium assessments are outlined in Fig. 1. Baseline demographics are presented in Table 1. Patients had a median age of 58 (IQR 47–68), and 171 patients (41% of the cohort) were admitted to the surgical ICU. The population was predominantly mechanically ventilated at enrollment (*n* = 350, 84%), median modified SOFA score was 7 (IQR 5–9), and Charlson Comorbidity Index was 2 (IQR 1–4). Many patients received benzodiazepines (*n* = 309, 74%) and opiates (*n* = 367, 88%). Delirium occurred in 308 (74%) patients during their ICU stay with a median duration of delirium of 3 (IQR 2–6) days. Antimicrobial exposure to any agent was common in around three fourths of subjects (*n* = 318, 76%), including the classes of beta-lactams (*n* = 223, 53%), fluoroquinolones (*n* = 138, 33%), macrolides (*n* = 29, 7%), and other antimicrobials (*n* = 285, 68%). One quarter (*n* = 100, 24%) of subjects did not receive any antimicrobials in their ICU stay. Patients were exposed to a wide variety of non-mutually exclusive antimicrobial agents, which are presented in Additional file 1: Table S1.

**Table 1:** Baseline demographics of critically ill cohort with medication records

Demographic	*N* = 418 (100%)^a^
Age, years	58 (47–68)
Sex, male	218 (52%)
Race, white	366 (88%)
Education level, years	12 (12–14)
ICU type	
SICU	171 (41%)
MICU	247 (59%)
Mechanical ventilation at enrollment	350 (84%)
Sepsis diagnosis at enrollment	116 (28%)
Modified SOFA score at enrollment	7 (5–9)
Charlson Comorbidity Index	2 (1–4)
Pre-existing cognitive impairment, IQCODE category	
Mild impairment	51 (12%)
Not impaired	367 (88%)
Delirium	
Patients with delirium	308 (74%)
Days of delirium	3 (2–6)
Coma	
Patients with coma	234 (56%)
Days of coma	2 (1–4)
Use of antimicrobials in the ICU	318 (76%)
Use of beta-lactams in the ICU	223 (53%)
Use of fluoroquinolones in the ICU	138 (33%)
Use of macrolides in the ICU	29 (7%)
Use of other^b^ antimicrobials in the ICU	286 (68%)
Use of antimicrobials in the ICU, days^c^	
First- to third-generation cephalosporins	2 (1–3.8)
Cefepime	4.5 (3–7)
Penicillins	4 (2–6)
Carbapenems	5 (3–8)
Fluoroquinolones	3 (2–5)
Macrolides	2 (1–6)
Other antimicrobials^b^	5 (3–9.8)
Use of analgesics and sedatives in the ICU	
Benzodiazepine	313 (75%)
Propofol	156 (37%)
Dexmedetomidine	88 (21%)
Opiates	368 (88%)
Use of antipsychotic in the ICU^d^	
Typical antipsychotic	117 (28%)
Atypical antipsychotic	102 (24%)

*Abbreviations*: *ICU* intensive care unit, *IQCODE* The Informant Questionnaire on Cognitive Decline in the Elderly, *MICU* medical intensive care unit, *SICU* surgical intensive care unit, *SOFA* Sequential Organ Failure Assessment (excluding neurologic component)

^a^All values presented as number (percentage) or median (interquartile range)

^b^For other antimicrobial list, see Additional file 1**:** Table S1

^c^*N* = 318

^d^Typical antipsychotic (for example, haloperidol), atypical antipsychotic (for example, quetiapine and olanzapine)

Among antimicrobial classes, only first- through third-generation cephalosporins demonstrated an association with delirium in the main statistical model (Table 2) (logistic regression with cluster sandwich covariance estimator model OR = 2.2, 95% CI 1.28–3.79, *P* = 0.004). This was corroborated on the sensitivity analyses restricted to ICU time accounting for daily sepsis and daily mechanical ventilation in Additional file 1: Table S2. Even after we adjusted for ICU type (that is, surgical versus medical) in our main statistical model and sensitivity analysis, our findings remained consistent. The classes of cefepime, penicillins, carbapenems, fluoroquinolones, and macrolides did not have any consistent associations with delirium across our models. After we accounted for total antimicrobial exposure in the second statistical model (Table 3), only the other antimicrobial class demonstrated an association between delirium and total days of exposure (proportional odds logistic regression model, OR = 3.14, 95% CI 2.27–4.35, *P* <0.001). The beta-lactams, fluoroquinolones, and macrolides did not have an association between total antimicrobial exposure and days of delirium.

**Table 2:** Delirium risk after antimicrobial exposure using a logistic regression model with cluster sandwich covariance estimator

Independent variable	OR	95% CI	*P* value
Antimicrobials on previous day			
Beta-lactams			
First- to third-generation cephalosporins	2.20	1.28–3.79	0.004
Fourth-generation cephalosporins	0.97	0.68–1.38	0.867
Penicillins	1.26	0.98–1.62	0.067
Carbapenems	1.43	0.97–2.11	0.069
Fluoroquinolones	0.92	0.69–1.23	0.572
Macrolides	0.64	0.29–1.43	0.274
Other antimicrobials^a^	1.44	1.15–1.79	< 0.001
Covariates	OR	95% CI	P-value
Age at enrollment	1.50	1.31–1.71	< 0.001
Mechanical ventilation at enrollment	2.03	1.38–3.00	< 0.001
Sepsis at enrollment	1.18	0.93–1.5	0.182
Modified SOFA score, same day	1.04	0.92–1.18	0.494
Charlson Comorbidity Index	1.07	0.91–1.26	0.391
IQCODE score	1.00	0.98–1.03	0.769
Delirium on previous day	11.21	9.41–13.35	< 0.001
ICU type, surgical	1.02	0.82–1.27	0.859
Dose of analgesics and sedativess^b^ on previous day			
Daily dose of benzodiazepines, mg^c^	1.16	1.06–1.25	< 0.001
Daily dose of propofol, mg	2.24	1.15–4.37	0.017
Daily dose of dexmedetomidine, μg	3.90	1.32–11.54	0.014
Daily dose of opiates, μg^d^	1.19	1.03–1.37	0.015
Use of antipsychotic on previous day^e^			
Typical antipsychotic	1.62	1.15–2.29	0.006
Atypical antipsychotic	1.35	1.07–1.69	0.012

*Abbreviations*: *CI* confidence interval, *ICU* intensive care unit, *IQCODE* The Informant Questionnaire on Cognitive Decline in the Elderly, *OR* odds ratio, *SOFA* Sequential Organ Failure Assessment (excluding neurologic component)

^a^For other antimicrobial list, see Additional file 1: Table S1

^b^Analgesic and Sedative drug doses were cube root transformed to reduce the influence of extreme outliers

^c^Midazolam equivalents, for example, midazolam 2.5 mg = lorazepam; 1 mg = diazepam 5 mg

^d^Fentanyl equivalents, for example, fentanyl 100 μg = hydromorphone 0.75 mg = morphine 5 mg

^e^Typical antipsychotic (for example, haloperidol), atypical antipsychotic (for example, quetiapine and olanzapine)

**Table 3:** Delirium risk after antimicrobial exposure using a proportional odds logistic regression model

Independent variable	OR	95% CI	*P* value
Total days of antimicrobial therapy			
Beta-lactams			
First- to third-generation cephalosporins	4.61	0.78–27.11	0.091
Fourth-generation cephalosporins	1.83	0.28–12.11	0.533
Penicillins	1.08	0.90–1.29	0.404
Carbapenems	2.14	0.51–9.04	0.299
Fluoroquinolones	1.05	0.89–1.24	0.566
Macrolides	0.16	0.03–1.03	0.054
Other antimicrobials^a^	3.14	2.27–4.35	< 0.001
Covariates	OR	95% CI	p-value
Age at enrollment	1.79	1.39–2.3	< 0.001
Mechanical ventilation at enrollment	4.75	2.51–9.0	< 0.001
Sepsis at enrollment	0.84	0.55–1.28	0.416
Modified SOFA score, same day	1.16	0.90–1.48	0.255
Charlson Comorbidity Index	0.88	0.69–1.14	0.344
IQCODE score	1.05	0.99–1.12	0.129
ICU type, surgical	0.86	0.57–1.30	0.477
Use of analgesics and sedatives in the ICU			
Benzodiazepines	1.03	0.68–1.59	0.875
Propofol	0.86	0.57–1.30	0.472
Dexmedetomidine	0.55	0.21–1.45	0.227
Opiates	1.03	0.6–1.77	0.904
Use of antipsychotics in the ICU^b^			
Typical antipsychotic	1.28	0.49–3.32	0.613
Atypical antipsychotic	2.03	0.7–5.94	0.194

*Abbreviations*: *CI* confidence interval, *ICU* intensive care unit, *IQCODE* The Informant Questionnaire on Cognitive Decline in the Elderly, *OR* odds ratio, *SOFA* Sequential Organ Failure Assessment (excluding neurologic component)

^a^For other antimicrobial list, see Additional file 1: Table S1

^b^Typical antipsychotic (for example, haloperidol), atypical antipsychotic (for example, quetiapine and olanzapine)

The most consistent remaining risk factors (main and sensitivity analyses) ranked from strongest to weakest were as follows: delirium on previous day, mechanical ventilation, sepsis, and age (Table 2 and Additional file 1: Table S2). Although other antimicrobial agents, most analgesic and sedative agents, and antipsychotics were associated with risk of delirium, the association was not significant after we adjusted for daily sepsis and daily mechanical ventilation in the ICU model (Additional file 1: Table S2**)**.

## Discussion

We found that first-, second-, and third-generation cephalosporins doubled the odds of delirium after adjusting for baseline co-morbidities, the course of critical care, and other competing antimicrobials and psychotropic medications risks. However, we did not find an association between total days of exposure and delirium in our proportional odds logistic regression model. We also did not find an association between delirium and cefepime. This is the first and largest nested cohort examining the association of antimicrobials and a rigorous longitudinally measured outcome for delirium.

Beta-lactam–induced neurotoxicity is widely recognized, specifically related to penicillins and cephalosporins [2–5, 11, 12, 31–36]. In addition to delirium (often termed encephalopathy [2]), reports of beta-lactam–induced neurotoxicity identify a variety of specific clinical features, including convulsive seizures, non-convulsive status epilepticus, aphasia, and myoclonus. Neurotoxicity may also be more pronounced in elderly patients, patients with renal insufficiency, and patients with prior neurologic disease who are more prone to neurotoxic effects; however, many of these studies were improperly powered or structured to examine these associations and without comparisons to those exposed to other antimicrobials or no antimicrobials. Our study focused specifically on delirium while addressing and adjusting for age, fluctuation in renal function as indicated by the daily modified SOFA score, pre-existing cognitive impairment, and a control sample of nearly 25% of subjects unexposed to any antimicrobial. Although other neurotoxicity features were beyond the scope of our work, we would remark that seizures occurred infrequently in our cohort (12 patients or 3%), where delirium was observed much more commonly (308 patients or 74%).

Available data previously suggested that, among the beta-lactam antibiotics, cefepime, which is commonly initiated in critically ill patients, might carry the highest risk of mental status change and encephalopathy [2, 3]. A previous retrospective study of 100 patients identified that cefepime neurotoxicity occurred in 15%, and likelihood of causality was ascribed via a modified Delphi method [3]. In the same study, the clinical dose of cefepime was appropriately adjusted for renal clearance in 64 patients (75.3%) without cefepime neurotoxicity and four patients (28.6%) with neurotoxicity (*P* = 0.001). Based on findings of retrospective studies like this, cefepime has been clinically identified as a reversible and potentially under-recognized cause of delirium in the ICU, particularly in those patients with renal failure. Clinicians have used this data to justify discontinuation of cefepime after encountering non-seizure mental status change [37].

In comparison with previous studies, we used a reliable and validated tool for acute brain dysfunction monitoring that is employed worldwide and translated in over 30 languages: the CAM-ICU [26]. All patients in our study, again in contrast to prior investigations, were under the co-management of an ICU pharmacist dedicated to renal dose adjustments daily. In order to control for time-varying elements of critical illness and organ failure (that is, renal impairment), our regression analysis used a daily modified SOFA score. Daily modified SOFA score was not a consistent risk factor for delirium identified in this study; therefore, other factors of critical illness may have had a more significant impact on risk of delirium in this population rather than the presence of organ dysfunction alone. Our nested cohort data demonstrate that isolated delirium in the setting of cefepime exposure does not necessarily imply causation or require discontinuation (in the absence of other neurotoxicity) given its lack of association with next-day delirium in any of our regression models, including restricting observation to the sickest patients [22].

Our study has several strengths. There is a limited body of literature regarding the association between various antimicrobial classes and delirium in critically ill patients. The current body of literature lacks utilization of a validated monitoring tool such as the CAM-ICU [38] to assess for presence of delirium in critically ill patients in accordance with the 2018 Society of Critical Care Medicine's Clinical Practice Guidelines for the Prevention and Management of Pain, Agitation/Sedation, Delirium, Immobility, and Sleep Disruption in Adult Patients in the ICU [39]. Our nested cohort study is the first to assess the association between antimicrobial administration and risk of delirium through longitudinal assessments of the CAM-ICU. Additionally, our study included both medical and surgical ICU patients who received any antimicrobial agent, which improves real-world generalizability, and sought to identify a specific class of agents associated with delirium. Post-operative critically ill adults may be at a particularly high risk for delirium [40]. After we adjusted for ICU type, our findings remained consistent in our main statistical model and sensitivity analysis. Furthermore, this study used a longitudinal statistical model in order to adjust for ICU-related risk and confounders for delirium on a daily basis as well as the temporal change in both exposure and outcome. Even in our most restrictive model isolated to critical illness, where time-varying covariates of sepsis and mechanical ventilation were known daily, our findings remained consistent. Lastly, our study employed a proportional odds logistic regression model in order to measure the association between total antimicrobial exposure and delirium. Pharmacokinetic and pharmacodynamic properties vary among different classes of antimicrobial agents, and total antimicrobial exposure and accumulation may have an impact on the outcome of delirium. Although this model did not identify an association between total exposure of first-, second-, and third-generation cephalosporins and total days of delirium, this model is limited by onset and duration of delirium. Delirium is a fluctuation in mental status that can change over time and may have occurred prior to or after antimicrobial therapy.

This is the largest evaluation thus far to examine the association between antimicrobial administration and risk of delirium in a critically ill patient population; however, limitations include the single-center population because of the inability to obtain antimicrobial data from the second institution, lack of adjustment for more subtle indicators of hepatic or renal dysfunction (or both), unknown microbial confounding, antimicrobial indication and susceptibility patterns, and unknown reason for admission to the ICU. Additionally, the effects of combination therapy and antimicrobial intensity therapy were not evaluated. Specifically, although the BRAIN-ICU study had only seven subjects with a primary neurologic diagnosis and none of these had sepsis on admission, we cannot confirm whether later infectious diagnoses included encephalitis or meningitis (or both) in any aspect of the cohort. It is unknown whether any providers prescribing antimicrobials shifted agents when encountering delirium. Also, when delirium occurs, reversible causes like seizures are considered part of our differential diagnosis [41], but electroencephalography is not applied to every patient.

Although this study fills an important void in the literature, antibiotics used in the time frame in which it was conducted (between March 2007 and May 2010) may have have been different from those of the present day. We are limited by the different toxicity profiles of each antimicrobial agent and clearance mechanisms that may be better assessed by using a continuous delirium metric. However, none currently exists, so we were constrained by our observations of outcome (that is, delirium measured twice daily in the ICU). There are many other antimicrobials used worldwide within the classes and subclasses that we studied; therefore, our patients were not exposed to every antimicrobial available and they were not exposed in similar distributions to every class.

This study adjusted for many ICU-related risks and confounders for delirium on a daily basis, however the presence of hypertension, all potentially deliriogenic medications such as steroids, and blood transfusions both which have been associated with delirium were not accounted for. The number of patients who received an electroencephalogram and the results of these was not collected. The incidence of delirium in the study population was high but appropriate for the study time frame. This study was conducted prior to the 2013 Pain, Agitation, and Delirium Guidelines that recommend non-benzodiazepine sedation and prior to the launch of the ICU Liberation ABCDEF Bundle Improvement Collaborative aiming to foster the bedside application of the PAD guidelines [42]. Our sample excluded patients with suspected severe baseline neurologic disease or admission for serious neurologic disease (for example, stroke and traumatic brain injury); thus, findings may not be extrapolated to this patient population in the setting of overt blood–brain barrier damage, although our multinomial models did adjust for the possibility of coma.

Further studies are now needed to examine mechanisms underlying the association between first-, second-, and third-generation cephalosporins and risk of delirium. This research could include pharmacokinetic and pharmacodynamic studies, accounting for differential blood–brain barrier penetration, utilization of antimicrobial monitoring when available, the role of gamma-aminobutyric acid (GABA) receptor agonism/antagonism [37], and current definitions of sepsis [43]. Patients with neurological injuries were excluded from this study. Given that many patients in a neurology ICU receive high-dose ceftriaxone for central nervous system infections, future studies need to be conducted within this patient population. Additionally, other antimicrobial agents and some analgesics, sedatives, and antipsychotics are associated with risk of delirium in the primary analysis only and not in the sensitivity analysis restricted to time in the ICU. There are multicenter randomized trials studying delirium in critically ill patients and the roles of antipsychotics (http://clinicaltrials.gov/ct2/show/NCT01211522; typical versus atypical) [44] and sedatives (https://clinicaltrials.gov/ct2/show/NCT01739933; propofol versus dexmedetomidine in sepsis). These trials may shed further light on these complex issues in critically ill patients, who are concomitantly exposed to antimicrobial agents. Our data may suggest that ICU-related risks, such as mechanical ventilation and the previous day’s mental status, may have a more profound impact on current mental status and delirium than simply antimicrobial exposure alone.

## Conclusions

In critically ill adult patients with respiratory failure or shock or both, we found that the OR for delirium doubled when patients were exposed to a wide group of first-, second-, and third-generation cephalosporins but not to penicillins, carbapenems, cefepime, fluoroquinolones, or macrolides. Future studies need to be conducted to determine the effect of different classes of antimicrobials on delirium and, in particular, of newer antibiotics, whose use has increased in recent years with the development of multidrug-resistant organisms. At this time, clinicians should remain hesitant about reflexively shifting active antimicrobial strategies simply because of the occurrence of delirium.

## Additional file

Additional file 1: **Table S1.** Antimicrobial agents used in the critically ill cohort (organized by major class and frequency). **Table S2.** Delirium risk after antimicrobial exposure using a logistic regression model with cluster sandwich covariance estimator restricted to intensive care unit (ICU) days. (DOCX 46 kb)

## Abbreviations

BRAIN-ICU: Bringing to Light the Risk Factors and Incidence of Neuropsychological Dysfunction in Intensive Care Unit Survivors
CAM-ICU: Confusion Assessment Method for the intensive care unit
CI: Confidence interval
ICU: Intensive care unit
IQCODE: Short Informant Questionnaire on Cognitive Decline in the Elderly
IQR: Interquartile range
OR: Odds ratio
PAD: Pain, Agitation, and Delirium
SOFA: Sequential Organ Failure Assessment
