# Supplementary material for: Antimicrobial exposure and the risk of delirium in critically ill patients
Source: Crit Care. 2018 Dec 12;22:337. doi: 10.1186/s13054-018-2262-z (PMC6291937; doi:10.1186/s13054-018-2262-z)
Supplement: Supplementary file 1 — Table S1. Antimicrobial agents used in the critically ill cohort (organized by major class and frequency). Table S2. Delirium risk after antimicrobial exposure using a logistic regression model with cluster sandwich covariance estimator restricted to intensive care unit (ICU) days. (DOCX 46 kb) [file 13054_2018_2262_MOESM1_ESM.docx]

**Table S1.** Antimicrobial Agents Used in Critically Ill Cohort (Organized by Major Class and Frequency)

| Antimicrobial Therapy | N=318 (100%) |
| --- | --- |
| Beta-lactams |  |
| 1st Generation Cephalosporins |  |
| Cefazolin | 10 (2.4%) |
| Cephalexin | 2 (0.5%) |
| 2nd Generation Cephalosporins |  |
| Cefoxitin | 1 (0.2%) |
| 3rd Generation Cephalosporins |  |
| Ceftriaxone | 24 (5.7%) |
| Cefotaxime | 3 (0.7%) |
| 4^th^ Generation Cephalosporin |  |
| Cefepime | 64 (15%) |
| Penicillins |  |
| Piperacillin-tazobactam | 117 (28%) |
| Ampicillin-sulbactam | 28 (6.7%) |
| Nafcillin | 8 (1.9%) |
| Penicillin G | 5 (1.2%) |
| Ampicillin | 5 (1.2%) |
| Amoxicillin-clavulanate | 4 (1.0%) |
| Amoxicillin | 2 (0.5%) |
| Ticarcillin-clavulanate | 1 (0.2%) |
| Carbapenems |  |
| Imipenem-cilastatin | 28 (6.7%) |
| Meropenem | 26 (6.2%) |
| Ertapenem | 22 (5.3%) |
| Fluoroquinolones |  |
| Levofloxacin | 117 (28%) |
| Ciprofloxacin | 34 (8.1%) |
| Moxifloxacin | 1 (0.2%) |
| Macrolides |  |
| Azithromycin | 23 (5.5%) |
| Erythromycin | 7 (1.7%) |
|  |  |
|  |  |

**Table S1 (continued).** Antimicrobial Agents Used in Critically Ill Cohort (Organized by Major Class and Frequency)

| Antimicrobial Therapy | N=318 |
| --- | --- |
| Other |  |
| Vancomycin | 228 (54.5%) |
| Antifungals^2^ | 200 (47.9%) |
| Antivirals^3^ | 84 (20.1%) |
| Metronidazole | 81 (19.4%) |
| Aminoglycosides^4^ | 76 (18.2%) |
| Linezolid | 54 (12.9%) |
| Sulfamethoxazole-trimethoprim | 43 (10.3%) |
| Antiretrovirals^5^ | 23 (5.5%) |
| Clindamycin | 13 (3.1%) |
| Rifampin | 12 (2.9%) |
| Doxycycline | 11 (2.6%) |
| Tigecycline | 7 (1.7%) |
| Dapsone | 8 (1.9%) |
| Pentamidine | 5 (1.2%) |
| Isoniazid | 1 (0.2%) |
| Pyrazinamide | 1 (0.2%) |
| Ethambutol | 1 (0.2%) |

^1^Data presented as n (%)

^2^Antifungals: Fluconazole, Amphotericin B, Itraconazole, Micafungin, Posaconazole, Voriconazole

^3^Antivirals: Acyclovir, Valacyclovir, Ganciclovir

^4^Aminoglycosides: Amikacin, Gentamicin, Tobramycin

^5^Antiretrovirals: Abacavir, Abacavir-Lamivudine-Zidovudine, Atazanavir, Darunavir, Lamivudine, Lamivudine-Zidovudine, Lopinavir-Ritonavir, Raltegravir, Ritonavir, Tenofovir, Zidovudine

| **Independent Variable** | **OR** | **95% CI** | **P-value** |
| --- | --- | --- | --- |
| Antimicrobials on Previous Day |  |  |  |
| Beta-lactams |  |  |  |
| 1^st^-3^rd^ Generation Cephalosporins | 2.13 | 1.1-4.1 | 0.024 |
| 4^th^ Generation Cephalosporins | 1 | 0.65-1.53 | 0.995 |
| Penicillins | 1.02 | 0.79-1.32 | 0.864 |
| Carbapenems | 1.19 | 0.80-1.78 | 0.394 |
| Fluoroquinolones | 0.89 | 0.65-1.21 | 0.447 |
| Macrolides | 0.61 | 0.25-1.52 | 0.291 |
| Other antimicrobials^1^ | 0.98 | 0.75-1.29 | 0.904 |
| **Covariates** | **OR** | **95% CI** | **P-value** |
| Age at Enrollment | 1.41 | 1.19-1.67 | <0.001 |
| Mechanical ventilation use, Same Day | 2.21 | 1.66-2.93 | <0.001 |
| Sepsis occurrence, Same Day | 1.79 | 1.40-2.3 | <0.001 |
| Modified daily SOFA score, Same Day | 0.87 | 0.73-1.02 | 0.090 |
| Charlson Comorbidity Index | 1.08 | 0.95-1.23 | 0.227 |
| IQCODE score | 1.03 | 0.98-1.08 | 0.192 |
| Delirium on Previous Day | 7.01 | 5.64-8.71 | <0.001 |
| ICU Type, Surgical | 1.34 | 1.02-1.77 | 0.037 |
| Dose of Analgesics and Sedativess^2^ on Previous Day |  |  |  |
| Daily dose of benzodiazepines (mg)^3^ | 0.97 | 0.83-1.13 | 0.707 |
| Daily dose of propofol (mg) | 1.41 | 0.79-2.52 | 0.241 |
| Daily dose of dexmedetomidine (mcg) | 1.6 | 0.58-4.46 | 0.365 |
| Daily dose of opiates (mcg)^4^ | 0.97 | 0.78-1.22 | 0.819 |
| Use of Antipsychotic on Previous Day^5^ |  |  |  |
| Typical Antipsychotic | 1.47 | 1.02-2.11 | 0.036 |
| Atypical Antipsychotic | 1.13 | 0.85-1.51 | 0.408 |

**Table S2.** Delirium Risk after Antimicrobial Exposure using a Logistic Regression Model with Cluster Sandwich Covariance Estimator Restricted to ICU Days

Abbreviations: CI=Confidence Interval; ICU=Intensive Care Unit; IQCODE: The Informant Questionnaire on Cognitive Decline in the Elderly; OR= Odds Ratio; SOFA=Sequential Organ Failure Assessment (excluding neurologic component)

^1^For other antimicrobial list, see **Supplementary Table 1**

^2^Analgesic and Sedative drug doses were cube root transformed to reduce the influence of extreme outliers

^3^Midazolam equivalents, e.g., midazolam 2.5 mg= lorazepam; 1 mg= diazepam 5 mg

^4^Fentanyl equivalents, e.g., fentanyl 100 mcg= hydromorphone 0.75 mg= morphine 5 mg

^5^Typical antipsychotic (e.g. haloperidol), Atypical antipsychotic (e.g., quetiapine, olanzapine)
